# Supplementary material for: Are carbon emissions trading and green financial instruments synergistic? -Comprehensive quantitative research based on content analysis
Source: PLoS One. 2024 Mar 7;19(3):e0298601. doi: 10.1371/journal.pone.0298601 (PMC10919601; doi:10.1371/journal.pone.0298601)
Supplement: S1 Data — (ZIP) [file pone.0298601.s001.zip › Data/地级市Z_F工作报告.zip!/║╙▒▒/╩»╝╥╫»╩╨/└┤╫╘╣1⁄2╓┌║┼í╛┬φ┐╦╩2╛▌═°í┐_2016.docx]

各位代表：

现在，我代表市人民政府向大会作工作报告。从今年开始，我市将实施第十三个五年规划，根据市委九届七次全会精神，市政府制定了《石家庄市国民经济和社会发展第十三个五年规划纲要（草案）》。请各位代表连同《纲要（草案）》一并审议，并请市政协委员和列席会议的同志提出意见。

一、“十二五”时期工作回顾

“十二五”期间，面对错综复杂的国内外环境和艰巨繁重的改革发展任务，市政府在省委、省政府和市委的正确领导下，紧紧依靠全市人民，围绕转型升级、跨越赶超、建设幸福石家庄的总目标，深入实施中东西区域协调发展战略，全力以赴推进全市经济社会持续快速健康发展。特别是党的“十八大”以来，我们认真贯彻习近平总书记系列重要讲话精神，坚持稳中求进工作总基调，主动适应发展新常态，齐心协力，锐意进取，统筹推进稳增长、调结构、促改革、治污染、惠民生，较好地完成了“十二五”规划确定的主要目标任务，经济社会发展取得了新成就。

过去的五年，是经济持续快速增长，综合实力显著增强的五年。始终把加快发展作为第一要务，经济增长的质量和效益显著提升。预计2015年全市地区生产总值达到5350亿元，年均增长9.4%，占全省比重由2010年的16.7%提高到17.8%，人均生产总值达到5万元，比2010年增加1.6万元。全部财政收入达到778.5亿元，其中一般公共预算收入完成375亿元，年均分别增长15%、18%，占全省的比重分别由2010年的14.8%、12.4%提高到19.2%、14.1%。固定资产投资达到5720亿元，年均增长19%。社会消费品零售总额达到2680亿元，年均增长13.7%。

过去的五年，是结构调整力度不断加大，发展方式加快转变的五年。经济结构持续优化，三次产业比重由2010年的10.9∶48.6∶40.5调整到9.4∶45∶45.6。生产力布局日益优化。中东西区域协调发展战略深入实施，中部服务业增加值占全市比重达到71.5%，东部规模以上工业增加值占全市的比重达到42%，西部绿色屏障功能日益增强。工业强市战略成效显著。预计2015年全市规模以上工业增加值完成2117亿元，年均增长10.9%；利润达到790亿元，占全省的36%，年均增长13.8%。全市规模以上高新技术企业达到413家，增加值达到329亿元，年均增长18.5%。五年新增市级以上工程技术研究中心、重点实验室、企业技术中心338家，连续9次被评为全国科技进步示范市。服务业实现快速发展。商贸设施不断完善，成功列入国家电子商务示范城市、城市共同配送体系等10个国家级试点，电子商务、商贸物流、金融服务、文化旅游等现代服务业发展迅速，预计2015年服务业增加值完成2440亿元，年均增长10.5%。农业现代化步伐加快。粮食生产实现“十二连丰”，总产达到505万吨，蔬菜、林果、畜牧产业稳定发展，五年新增市级以上农业产业化龙头企业118家，农业产业化经营率达到65.5%，成功入选国家现代农业示范区。

过去的五年，是城镇建设上水平出品位，新型城镇化加速推进的五年。顺利完成部分行政区划调整，城区面积由469平方公里扩展到2206平方公里，“一河两岸三组团”城市发展新格局初步形成。交通路网日臻完善。正定国际机场第二航站楼建成投用，京石武高铁全面运营，石济客专正式开工。西柏坡高速、京港澳高速改扩建、京昆高速石太北线和307国道、308国道、新赵线等改建工程顺利完成。全市公路通车总里程达1.8万公里，比2010年增加2624公里。城市功能显著提升。新客站投入运营，东广场及周边路网连接贯通。轨道交通1、3号线30公里路段实现“洞通”。新改建太行大街、新城大道、新胜利大街等83条市区道路。集中实施了一批供热、供水、供电、通讯、地下管网改造等城市基础设施工程，城市承载能力明显提升。南水北调中线总干渠建成通水，配套工程建设扎实推进。正定新区建设步伐加快。累计完成投资198亿元，建成道路20条，“三纵三横”主路网框架基本形成，园博园、新区第一中学建成投用，特教学校、信息技术学校主体竣工，河北奥体中心、国际展览中心、综合商务中心等功能性设施加速推进。正定古城保护取得阶段性成效。城镇面貌明显改善。五年累计完成投资1365亿元，实施县城建设攻坚项目2160个。鹿泉、晋州、井陉、高邑、元氏荣获省人居环境进步奖，省级以上园林县城达到11个。深入开展美丽乡村建设，完成1213个省级重点村建设任务。全市常住人口城镇化率由2010年的50.8%提高到58%。

过去的五年，是改革开放向纵深推进，发展活力不断增强的五年。商事制度改革实现突破，“三证合一”、“一照一码”制度全面实施，市场主体总量突破56万户，比2010年增加25万户。国有企业改革成效明显，完成68家国有企业改制，2015年22家监管企业营业收入达到293.9亿元，年均增长11.8%。财政体制改革进一步深化，绩效预算管理全面推开，“营改增”政策深入实施。投融资体制改革取得积极进展，2015年新增贷款规模达到986.8亿元，贷存比达到63.8%，全市在各类资本市场挂牌上市的企业达到112家，其中“十二五”期间新增90家。农村综合改革步伐加快，土地承包经营权确权登记颁证420.9万亩，供销社改革经验在全省推广。对外开放不断扩大，综合保税区通过省预验收，石洽会、冀商大会、通用航空展等展会影响力不断扩大。五年实际利用外资48.3亿美元，引进市外资金4800亿元，外贸进出口总值完成680亿美元，对外投资达到17.6亿美元。协同发展实现良好开局，京津冀产学研联盟正式成立，与京津合作项目达到158个，总投资2529.4亿元。

过去的五年，是生态建设取得显著成效，人民幸福指数不断提高的五年。始终把打造良好的生态环境和生活环境作为重大民生工程，努力增强人民群众的幸福感。生态环境质量持续改善。大气质量实现明显好转，优良天数从2013年的43天提高到180天，PM2.5浓度下降42.2%。累计压减燃煤998万吨、炼铁产能158万吨、炼钢产能160万吨、水泥产能1850万吨，提前三年完成水泥过剩产能压减任务，提前一年实现“十二五”总量减排目标，为京津冀区域空气质量改善，实现“APEC蓝”、“阅兵蓝”做出了重要贡献。集中实施洨河、汪洋沟、磁河综合整治工程，主要河流出境断面水质基本达标。五年完成造林260万亩，森林覆盖率达到37.2%，荣获“国家森林城市”和“全国绿化模范城市”称号。民生保障水平明显提升。2015年各级财政用于民生的支出达到560.5亿元，占财政总支出的比重达到82.1%，比2010年提高20.2个百分点。城乡居民人均可支配收入分别达到28076元和11600元，年均增长10.1%和13%。五年累计解决287万农村人口饮水安全问题，实现22.7万扶贫对象稳定脱贫。社会保障体系不断完善。城镇职工基本养老保险和城镇基本医疗保险参保人数持续增加，连续十一年提高企业退休人员养老金待遇。养老服务业快速发展，千名老人拥有床位达到32张。五年开工安居工程16.7万套，全面完成保障性住房建设任务。城乡低保实现应保尽保。社会事业全面发展。教育事业均衡发展，22个县（市）区全部通过省义务教育基本均衡督导评估；山区教育扶贫经验在全国推广。文化事业日益繁荣，市县乡村四级公共文化服务网络更加健全。医药卫生体制改革任务圆满完成，在全省率先实现标准化村卫生室全覆盖。人口和计生工作全面加强，计生特殊家庭医养扶一体化服务保障机制更加健全。治安防控体系不断完善，社会大局和谐稳定。食品药品安全专项整治活动扎实推进，“党政同责、一岗双责”安全生产责任体系全面建立，安全生产形势持续好转，连续六次入选全国十大幸福城市。国防动员深入开展，组织举办抗战胜利70周年系列纪念活动，在荣获全国双拥模范城“七连冠”基础上，顺利通过了第八次考核验收。援藏援疆任务圆满完成。妇女儿童、民族宗教、外事侨务、残疾人、气象、档案、人防等各项事业都取得了新成就。

过去的五年，是政府职能加快转变，自身建设不断加强的五年。市县政府机构改革全面完成，行政管理体制改革深入推进，非行政许可审批和行政监管类别全部取消，行政审批事项五年减少79%。自觉接受市人大法律监督、政协民主监督和社会舆论监督，累计办理人大代表建议1864件、政协委员提案2957件，办结率均达100%。反腐倡廉力度不断加大，查处损害发展环境问题1637件，实施责任追究1949人，营造了风清气正、干事创业的良好氛围。

各位代表！回顾五年的改革发展历程，我们万众一心，开拓奋进，经受住了国际金融危机的巨大冲击，有效应对经济下行压力的严峻挑战，努力克服国内经济“三期叠加”的不利影响，取得了经济和社会事业发展新成就。这是省委、省政府和市委正确领导的结果，是全市广大干部群众共同奋斗的结果。在此，我代表市政府，向全市人民，向人大代表、政协委员，向各民主党派、工商联、人民团体和社会各界人士，向驻石解放军、武警官兵和政法干警，向中直、省直机关驻石单位，向关心支持石家庄发展的香港和澳门特别行政区同胞、台湾同胞、海外侨胞、国内外朋友，致以崇高的敬意和衷心的感谢！

在肯定成绩的同时，我们也清醒地认识到，我市经济社会发展中还存在一些突出矛盾和问题，主要是：经济发展的质量和效益还不高，经济增长动力仍处在转换之中，战略性新兴产业尚未形成有效支撑；创新驱动主动力不足，科技成果转化率较低；生态环境压力依然较大，资源环境约束加剧；省会辐射带动作用不强，在京津冀协同发展中的地位有待提升；县域经济整体实力不强，新型城镇化进程不快；民生改善任务繁重，基本公共服务保障能力和社会管理水平还需提高；干部队伍作风建设还需进一步加强，行政管理体制改革任务仍然艰巨，发展环境仍需进一步优化。我们一定高度重视这些问题，坚持问题导向，采取有力措施，切实加以解决。

二、“十三五”时期经济社会发展的战略任务

“十三五”时期，是我市率先全面建成小康社会的决胜阶段，是全面深化改革开放、积极融入京津冀协同发展的关键阶段，是转型升级、跨越赶超、建设幸福石家庄的加速阶段。展望“十三五”发展大势，世界经济在深度调整中缓慢复苏，新一轮科技革命和产业变革蓄势待发。我国经济步入增速更稳、结构更优、质量更好、效率更高的新常态，特别是国家实施京津冀协同发展战略，为我们承接北京非首都核心功能疏解和京津产业转移，打造京津冀城市群“第三极”提供了千载难逢的历史机遇。我市经过“十二五”的发展，经济基础更加雄厚，产业结构日趋优化，城市框架已经拉开，为实现新一轮又好又快发展奠定了坚实基础。尤其是省委、省政府高度关注省会的建设和发展，赵克志书记明确要求我们在贯彻“创新、协调、绿色、开放、共享”发展理念上走在全省前列，在发展战略性新兴产业上走在全省前列，在完善城市规划和城市功能，特别是加强大气污染治理，千方百计退出全国污染城市后十位上走在全省前列，在创建文明城市、文明行业、文明单位、争做文明市民上走在全省前列。张庆伟省长要求我们要在发展理念的转变方面形成共识，在推进供给侧结构性改革方面形成共识，在抢抓京津冀协同发展重大机遇方面形成共识。同时，省委、省政府将出台支持省会建设发展的重要意见，为我们率先全面建成小康社会提供了坚强保障。我们必须牢牢抓住机遇，再接再厉，乘势而上，奋力书写省会经济社会发展新篇章！

“十三五”时期我市经济社会发展的指导思想是：全面贯彻党的十八大和十八届三中、四中、五中全会精神，以马克思列宁主义、毛泽东思想、邓小平理论、“三个代表”重要思想、科学发展观为指导，深入贯彻习近平总书记系列重要讲话精神，坚持“四个全面”战略布局，坚持“创新、协调、绿色、开放、共享”的发展理念，抓住京津冀协同发展重大历史机遇，以提高经济发展质量和效益为中心，坚守发展、生态、民生三条底线，深入实施中东西三大区域协调发展战略，大力推进新型工业化、信息化、城镇化和农业现代化，全面深化改革，扩大对外开放，加快形成引领经济发展新常态的体制机制和发展方式，统筹推进经济建设、政治建设、文化建设、社会建设、生态文明建设，加快转型升级、跨越赶超、建设幸福石家庄步伐，努力打造京津冀城市群“第三极”，确保率先在全省全面建成小康社会。

“十三五”时期我市经济社会发展的主要奋斗目标，概括起来就是“两个前列”、“两个翻番”、“三个确保”。“两个前列”：全市综合经济实力位居全省前列，经济质量和效益位居全省前列。“两个翻番”：到2020年，生产总值比2010年翻一番以上，城乡居民人均可支配收入比2010年翻一番以上。“三个确保”：确保大气质量得到明显改善，确保京津冀城市群“第三极”作用凸显，确保率先在全省全面建成小康社会。主要预期量化目标是：生产总值年均增长7.5%，一般公共预算收入年均增长8%以上，城乡居民人均可支配收入年均增长8%以上，按期完成国家和省下达的环境治理目标任务。

实现“十三五”发展目标，必须认识新常态、适应新常态、引领新常态，以习近平总书记提出的“十个更加注重”为基本遵循，把推进供给侧结构性改革作为适应和引领新常态的重大创新，提高供给体系质量和效率，提高投资有效性，提高全要素生产率；必须牢牢抓住发展第一要务不动摇，把握协同发展、转型升级、又好又快工作主基调，深入实施创新驱动发展战略，加快新动能成长和传统动能提升，努力实现更高质量、更有效率、更可持续的发展，全力实现七大战略任务新突破：

（一）全方位融入京津冀协同发展，在厚植省会发展优势上实现新突破。充分发挥冀中南功能拓展区的龙头带动作用，全力打造功能完备的现代省会城市和京津冀城市群“第三极”。构建一体化综合交通网络。积极推进京石邯城际铁路、津石铁路、石衡沧黄城际铁路和太行山高速、津石高速、石衡高速等国省干线建设，不断加密与北京、天津及周边地区互联互通的交通网络。推进绕城高速以内的高速公路实行开放式收费，不断完善主城区快速路体系。提升正定国际机场功能，全力打造京津冀区域航空物流枢纽中心。积极推进京津石综合交通运输信息共享平台建设，尽快实现京津冀区域内交通“一卡通”、客运“一票制”和货运“一单制”。构建功能明确的承接平台。把“大正定新区”建设作为参与京津冀协同发展的“一号工程”，全力申办国家级新区，主动承接公共服务、行政事业机构、企业总部、研发创新中心等非首都功能疏解和京津高端产业转移。统筹高新技术开发区、经济技术开发区等国家和省级产业园区，积极承接京津装备制造、电子信息、生物医药、轻工食品等产业转移。构建协同创新共同体。围绕石保廊全面创新改革试验区建设，积极与京津联合创建一批国家级协同创新中心、工程研究中心和重点实验室。充分发挥石家庄京津冀产学研联盟和科技大市场作用，支持企业与京津企业、高校和科研院所开展协同创新。主动参与设立京津冀产业结构调整、科技成果转化、扶贫开发等基金，努力形成优势互补、分工协作、创新共赢的发展格局。

（二）建设全面创新型城市，在推进发展动力转换上实现新突破。深入实施创新驱动发展战略，营造创新创业良好氛围，激发大众创业、万众创新的活力。坚持以科技创新引领发展。强化企业创新主体地位，全力打造一批创新型领军企业、培育一批高新技术企业、壮大一批科技型中小企业。大力推动重点领域技术创新，围绕高端产业开展重点创新，推动产业向中高端迈进。力争到2020年，研发投入占GDP的比重达到2.6%，全市科技型中小企业达到8000家，科技进步贡献率达到58%。坚持以体制创新驱动发展。加快完善技术创新市场导向机制，推动经济发展从依靠要素驱动转换到依靠创新驱动的轨道上来。健全以增加科技人员收入为核心的科技成果转化激励机制，整合科技创新资金，加强知识产权保护，促进科技成果加快转化。实施创业导师制度，整合行业专家、优秀企业家等各类人才资源，为创业创新主体提供培训和咨询服务。坚持以政策创新保障发展。大力实施高端专业技术人才引进和知识更新工程，建立与经济社会发展相适应的人才培养和引进支撑体系。健全普惠性扶持政策，支持社会资本、市场主体和各类开发区建设创新创业服务平台。到2020年，新建20家院士工作站、5个高技能人才培训基地，力争培养10万名各行各业高技能人才。

（三）建立新型现代产业体系，在调整优化产业结构上实现新突破。坚持信息化、高端化、服务化发展方向，积极落实“中国制造2025”和“互联网+”行动计划，加快形成一二三产业融合发展的新格局。着力壮大战略性新兴产业。大力提升生物医药产业、新一代信息技术产业、高端装备制造业发展水平，积极支持节能环保、新材料、新能源等新兴产业发展。每年滚动实施重点战略性新兴产业项目100项以上，到2020年高新技术产业增加值占全市规模以上工业增加值的比重达到22%以上。着力推进传统产业改造升级。制定工业转型升级和“互联网+制造业”实施计划，推进新一代信息技术与制造业深度融合。鼓励优势企业通过资本、技术等整合重组，实现集团化、集约化发展。力争到2020年，规模以上工业增加值年均增长6.5%以上，主营业务收入超100亿元企业达到10家。着力发展现代高端服务业。围绕金融服务、商务服务、现代物流、信息服务等八大重点领域，继续实施三年行动计划，着力建设现代流通体制，推动生产性服务业向专业化和价值链高端延伸，生活性服务业向精细化和高品质转变，商贸流通企业向信息化、标准化、集约化发展。力争到2020年，服务业增加值占生产总值的比重达到53%。着力提升农业现代化水平。进一步优化现代农业布局，支持中部县（区）大力发展都市休闲农业、西部山区县着力发展生态农业、东部平原县（市）开展农业产业化经营。积极培育新型农业经营主体，加强农业龙头企业建设，推进多种形式适度规模经营，不断提高农业组织化、标准化、品牌化和产业化水平。到2020年全市家庭农场发展到1500家以上，实体农民合作社达到4000家以上。

（四）优化城市空间发展格局，在促进区域平衡发展上实现新突破。积极推进国家新型城镇化综合试点城市建设，强化省会地位和作用，努力形成集现代城市、特色城镇、美丽乡村于一体的城乡统筹发展新格局。推进中东西区域协调发展。按照“一核一带两轴三区”市域空间结构，以产业结构调整和区域协调发展为重点，做大做强中部城市经济，加快发展东部工业经济，力促西部绿色工业、生态农业和旅游业发展，在全市域形成功能布局合理，城镇化、工业化与生态建设相协调的空间结构。推进组团式都市区建设。按照“一河两岸三组团”城市发展格局，以建设智慧城市、生态城市、海绵城市为抓手，划定城市边界，建设城市隔离带，加快主城区提档升级步伐，大力提升城市吸引力和竞争力。按照新的区划调整方案，积极推进组团一体化发展，加快城市交通、市政设施、城市管理同城化进程。实施城区排水管网综合改造，消除城市建成区黑臭水体。推进城乡一体化发展。加快建立农业转移人口市民化成本分担机制、多元化可持续的投融资机制，进一步优化行政区划和创新行政管理，综合推进体制机制改革创新，到2020年常住人口城镇化率达到63%，户籍人口城镇化率达到52%。深入实施县城建设攻坚行动，提升县城建设质量和水平，打造有历史记忆、地域特征、宜居宜业的美丽县城。实行县域经济增比进位考核，发展壮大县域特色产业，支持有条件的县（市）争创全国百强县。加快建设美丽乡村，全面改善农村生产生活条件，到2020年基本实现美丽乡村建设全覆盖。

（五）打造宜居绿色家园，在改善生态环境上实现新突破。牢固树立绿水青山就是金山银山的基本理念，坚定不移地走绿色低碳循环发展之路。持续推进大气污染防治。大力优化能源结构，不断提高清洁能源占能源消耗的比重。减少煤炭消费总量，到2020年原煤在一次能源消费中的比重下降到65%，洁净煤使用率达到90%以上。推进钢铁、水泥、电力、焦化、石化、制药等重点行业节能减排。实施企业搬迁升级改造工程，到“十三五”末高排放企业全部迁出主城区。确保到2018年大气质量退出全国重点监测城市后十位，到2020年实现大气质量明显好转。推进水环境综合整治。加大水源涵养和水污染防治力度，落实最严格的水资源管理制度，严控用水总量、用水效率和水功能区限制纳污“三条红线”。大力开展滹沱河等重点河流综合改造工程，加快恢复河流生态系统。加强南水北调配套工程建设，用足用好引江水。推进绿色石家庄工程建设。围绕巩固提升国家森林城市、国家园林城市建设水平，深入实施太行山绿化、沿河绿化、环省会绿化等生态建设工程，到2018年完成植树造林300万亩，实现太行山宜林地带绿化全覆盖，到2020年全市建成区绿地面积达到1万公顷，森林覆盖率达到42.2%以上。

（六）开创改革开放新局面，在增强发展动力活力上实现新突破。坚持把深化改革、扩大开放作为推动发展的强大引擎，更加注重市场在资源配置中的决定性作用，更加注重高水平双向开放。积极承担改革试点任务。坚持各领域改革开放向先进省市看齐，大力推进自贸区和京津改革开放经验在我市复制推广，争取中关村国家自主创新实验区和天津滨海新区政策向我市延伸。围绕建设新型城镇化与城乡统筹示范区、京津冀生态环境支撑区等，大胆创新，先行先试，创造可复制的经验做法。扎实推进重点领域改革。完善国有资产管理体制，实现经营性国有资产集中统一监管，分类推进国有企业改革，完善现代企业制度，发展混合所有制经济，建立覆盖全部国有企业的国有资本预算管理制度。深化财政体制改革，建立全面规范、公开透明的预算制度。加快金融体制改革，着力提高金融服务实体经济的效率，努力扩大直接融资比重。深化农村综合改革，积极探索农村集体资产股份权能改革，建立健全农村产权交易流转市场。深化商事登记制度改革，建立“双随机”抽查机制，构建事中事后监管新模式。全面提升开放层级和水平。积极营造法制化、国际化、便利化外商投资环境，毫不动摇地鼓励支持和引导非公有制经济发展，到2020年民营经济增加值占生产总值的比重达到75%以上。拓展“走出去”新路径，建立石家庄“一带一路”贸易商联盟和智库合作联盟，精准组织优势产能推介和项目对接。力争实际利用外资年均增长5%以上。

（七）全力增进民生福祉，在建设惠及全民的幸福省会上实现新突破。着眼保基本、兜底线、促公平，补齐全面建成小康社会的短板，切实增强人民群众的获得感。坚定不移完成脱贫攻坚任务。把脱贫攻坚放在改善民生首要位置，加大财政投入，落实“六个精准”要求，实施“五个一批”行动计划，确保2018年前，赞皇、灵寿、行唐、平山4个贫困县全部摘帽，到2020年全面完成30.2万农村贫困人口脱贫任务。实施创业就业扶持工程。实行更加积极的就业政策、更加完善的创业扶持政策，多渠道开发就业岗位，建立起覆盖城乡的公共就业服务体系和城乡平等的就业制度。到2020年，全市城镇新增就业45万人，城镇登记失业率控制在4.5%以内。实施社会保障完善工程。推进健康石家庄建设，整合城镇居民基本医疗保险和新型农村合作医疗两项制度，建立统一的城乡居民基本医疗保险制度。推动医疗、医保、医药“三医联动”，逐步实现基本医保、大病保险、医疗救助、商业健康保险衔接配合，努力构建多层次医疗保障体系。逐步提高城乡社会救助标准，实行农村低保线与扶贫线“两线合一”。加快发展社会福利、慈善救济事业，推进居家养老、社区养老和机构养老协调发展，大幅增加养老服务设施。实施教育优先发展工程。提高学前教育普及水平，促进义务教育均衡发展，普及高中阶段教育，大力发展现代职业教育，推进高等教育内涵式发展。完善家庭困难学生资助体系，促进教育公平。到2020年学前三年毛入园率达到95%，九年义务教育巩固率达到99.9%。实施文化软实力提升工程。以提升城市文明程度和市民文明素质为主线，加强公共文化和体育基础设施建设，全面提高公共文化服务水平，争创全国文明城市。大力开展文化惠民活动，加大对农村、偏远山区等薄弱地区的文化设施建设的支持力度。培育壮大文化市场主体，鼓励社会资本进入文化产业，加强对外文化交流，打造优势文化品牌。到2020年，全市文化产业增加值占生产总值的比重超过5%。

各位代表!目标承载使命，任务考验担当。只要我们万众一心，苦干实干，就一定能够铸就发展新辉煌、率先实现全面小康！

三、2016年主要工作

今年是实施“十三五”规划的开局之年，做好全年工作必须以“五大发展理念”为引领，按照宏观政策要稳、产业政策要准、微观政策要活、改革政策要实、社会政策要托底的总体要求，战略上坚持稳中求进、战术上坚持全力攻坚，大力推进供给侧结构性改革，去产能、去库存、去杠杆、降成本、补短板，加快发展动能转换和质量效益提升，促进经济社会持续健康发展。主要预期目标是：地区生产总值增长7.5%左右，一般公共预算收入增长8%，服务业增加值增长10%，规模以上工业增加值增长6%，固定资产投资增长11%，社会消费品零售总额增长11%左右。城乡居民人均可支配收入均增长8%，居民消费价格指数控制在103以内。围绕实现上述目标，重点做好以下九项工作：

（一）以科技创新为主动力，推进全面创新型城市建设。深入实施创新驱动发展战略，激发全社会创新潜能和创业活力，使省会成为全省创新创业最活跃、先进要素最密集的城市。推进协同创新示范区建设。充分发挥环保产业发展研究院等6家产业研究院和石家庄京津冀产学研联盟的作用，与京津合作共建一批产业技术创新和科技成果孵化转化基地，年内力争建设1家国家级协同创新中心，2个协同创新平台。积极推动科技大市场与中国技术交易市场、北方技术交易市场等建立统一报价系统，全年来自京津的技术合同交易额增长10%以上。大力培育创新型领军企业。实施高新技术产业倍增和科技型中小企业成长计划，重点支持20家创新型领军企业开展关键技术攻关，集中实施110项重大科技专项，研发推出省级以上新技术、新产品120项。加大政策扶持力度，设立2亿元市级融资支持资金，力促科技型中小企业做大做强，年内新增科技型中小企业600家、科技小巨人企业230家，新认定高新技术企业50家。努力打造高端创新平台。重点抓好东旭集团平板显示玻璃技术工程实验室、以岭药业创新中药重点实验室等重大创新平台建设，全年新建市级以上重点（工程）实验室、工程（技术）研究中心、企业技术中心30家。着力推进中关村创业大街、北大科技园、腾讯众创空间等创新创业载体建设，年内全市各类孵化载体达到50家以上，孵化器面积达到200万平方米、在孵企业达到1500家。激发大众创业万众创新。大力支持入选国家“千人计划”、“长江学者”等人才带项目来我市创业。鼓励发展众创、众包、众扶、众筹创新模式，建成一个服务功能完善的创新创业园，规范化、标准化众创空间达到10家以上，新认定科技领军人才15名，新增创新创业团队200个、创客1600名。优化人才政策，着力解决中低端人力资源同质化、高端和复合型人才不足问题。鼓励设立科技支行、科技担保、科技保险等专营服务机构，新增创投基金2家以上。

（二）以重点领域率先突破为发力点，深度融入京津冀协同发展。立足国家和省赋予的功能定位，推动京津冀协同发展战略在我市全面落实。申报建设国家级正定新区。以构建京津冀协同发展重要支撑区为目标，加大国家级正定新区的申报力度，力争早日获批。加快综合保税区建设，全面完成口岸设施、综合服务中心、信息化系统等设施建设，确保顺利通过封关验收。着力推动交通互联互通。启动京石邯城际铁路前期工作，加快南绕城高速建设，确保2017年建成通车。积极采取PPP模式，同时争取国家政策支持，力争平赞、西阜、津石、石衡高速早日开工建设，实现县县通高速、县县通一级公路目标。加大产业项目引进力度。启动总投资50亿元的北大科技城项目，以此为载体，建设功能完善、体系健全、京津资源聚集的科技创新基地。加大新兴产业对接合作力度，全年落地京津冀协同发展项目达到50项，力促大唐电信、翼辰铁科等一批合作项目竣工投产。

（三）以工业经济转型升级为主攻方向，推动产业向中高端迈进。推进工业强市战略向纵深发展，促进新兴产业加速壮大，全面提升工业经济质量和效益。加快提升现代医药产业。依托重点园区、龙头企业，积极推进企业向园区集聚，集中实施石药抗肿瘤系列新药产业化、常山生化生物原料药生产基地等总投资167亿元的25个重大项目，重点发展新型高端制剂、现代生物技术和现代中药，加快原料药绿色化、制剂高端化、中药国际化步伐。培育壮大新一代信息产业。依托中电科、四方通信等骨干企业，大力推进北斗卫星导航、有源光缆、LED光电产业化等总投资196亿元的21个重大项目建设，促进新一代电子信息产业加快发展，全面提升信息产业的规模和比重。积极培育大数据、云计算、物联网等新兴产业，构建石家庄大数据云平台，着力打造大数据全产业链。改造提升先进装备制造业。依托中航通飞、中车石家庄公司、格力电器等优势企业，实施中航通飞产业基地等总投资103亿元的14个重大项目，壮大城市轨道交通装备、通用航空特种装备、智能农业机械、家用服务机器人产业，提升高端装备能级水平。加快发展节能环保产业。重点支持中博、中车等新能源汽车加快发展，提高公交车、公务用车、私人用车中新能源汽车比例。鼓励节能环保等新兴产业发展，支持益生环保、英凯再生资源综合利用等重点企业做大做强，抓好井陉太科太阳能发电、柏森生物质燃料等新能源项目建设。推进传统产业转型升级。积极开展精准对标，把对标行动向县域产业集群延伸。围绕做优做强食品、纺织、石化等传统优势产业，滚动实施君乐宝乳业婴幼儿配方奶粉建设、常山集团新型纺织品开发等重点技改项目200项，技改投资增长10%以上。推进工业化与信息化深度融合，年内新培育省级“两化融合”重点企业10家以上。加大对实体经济的帮扶力度。开展降低实体经济企业成本行动，最大程度降低制度性交易成本、人工成本、企业税费负担、社会保险费、财务成本、电力价格、物流成本。设立中小微企业发展基金，鼓励企业发行债券、短期融资债券，努力拓展企业融资渠道。帮助规模以下企业提档升级，年内新增规模以上工业企业100家。通过资产重组、产权转让、关闭破产等方式，加快推进“僵尸企业”退出市场。鼓励民间资本进入更多领域，推动民营企业科技创新、调整结构、培树品牌，激发民营经济的活力和创造力。

（四）以发展现代服务业为龙头，力促城市经济上台阶。继续实施服务业重点领域三年行动计划，力争在拓展服务业领域、扩大服务业规模、提高服务业比重等方面取得突破性进展。做强金融服务业。精准做好银企对接工作，建立实体经济与金融机构常态化沟通机制，全市新增贷款规模继续保持全省领先。加强企业上市辅导培育，确保新增各类上市企业30家以上。加快设立石家庄农村商业银行，推进县级联社改制为农村商业银行。积极引导保险机构投资我市经济社会建设。提升现代物流业。以建设国家物流枢纽城市、城市共同配送国家试点为抓手，全力推进深国际、卓新物流、冀中南智能物流港、环城国际汽车物流园等18个重点项目建设，确保神威医药物流园一期等项目竣工投用。拓展信息服务业。提升信息服务基础设施水平，加快云计算产业园建设，积极培育大数据产业。加快建设“商务云+中国梦网”平台，支持北国如意购物网、慧聪大宗商品交易中心等电商平台扩大交易规模。推动4G网络向农村延伸，抓好正定、行唐等国家级电子商务进农村综合示范县建设，年内实现农村电子商务全覆盖。全年电商交易额达到2500亿元以上。大力发展会展业。加快国际展览中心建设，按照每半月举办一次大型会展、每季度举办一次国际性会展的目标，不断提升石洽会、旅交会、药博会、动博会等传统展会水平，举办好第二届中华健康节、“爱飞客”飞行大会等新兴展会，吸引更多国内外人士了解石家庄、投资石家庄。优化休闲旅游业。以打造环省会、西部山区旅游产业带为重点，加快精品景点、农家乐、旅游交通标识等基础设施建设，整合旅游资源，推出一批适合市民节假日“下乡进山”的精品线路。支持中山国遗址公园开发保护，打造古文化旅游品牌。落实带薪休假制度，鼓励错峰休假，有效促进旅游消费，力争全年实现旅游业总收入585亿元，同比增长15%。壮大文化教育体育产业。以发展职教培训为重点，整合职业教育资源，加快职教园区建设，推进产教深度融合、“中高专本”衔接融通。以做大做强影视制作、动漫游戏、出版发行等为目标，培育壮大市场主体，努力繁荣文化市场。加快体育基础设施建设，组织好“中超”主场比赛，争办全国性、国际性赛事，带动全市体育产业快速发展。加快发展健康养老业。全面放开养老市场，鼓励社会资本投资养老服务业。大力发展“医养结合”养老服务，支持中节能健康城、富力疗养度假中心、绿城养老养生园等重点项目建设，发展“全链式”健康养老服务业。提档升级传统服务业。支持北国、勒泰、万达等大型商贸零售企业加快转型发展，利用新型商业模式改造提升新华集贸、南三条等大型批发市场。高标准建设商贸流通服务设施，重点抓好华润万象综合体、北人集团奥特莱斯等大型商贸综合体项目。

（五）以“三农”工作为重中之重，加快县域小康建设步伐。构建现代新型农业产业体系，补齐县域经济发展短板，推动农业强、农村美、农民富。加快发展现代农业。围绕国家现代农业示范区建设，继续抓好50个小麦、48个玉米万亩高产示范片建设，粮食总产稳定在440万吨。发展壮大畜牧、蔬菜、林果三大优势产业，着力做大做强君乐宝、三元、双鸽3个省级畜牧业链条经济，新建30个市级蔬菜标准园、15个现代果品示范区。积极推广“葫芦峪”模式，建成13个市级以上现代农业园区。积极培育各类农业新型经营主体，推进适度规模经营，规范农村专业合作社注册管理，新发展家庭农场220家，实体农民合作社300家。大力发展县域经济。继续实施县（市）区党政一把手引进5亿元、10亿元以上项目责任制，加快引进和落地一批强县立县的大项目、好项目。支持晋州纺织、赵县生物制药、无极皮革等产业集群扩大规模、规范发展，力争主营业务收入超50亿元的产业集群达到20家。加大对县域发展的考核力度，改进考核办法，科学设置权重，激励各县（市）区争先进位，开创县域经济发展新局面。推进美丽乡村建设。以“四美五改”为重点，完成406个重点村建设任务，重点打造鹿泉抱犊寨、栾城三苏都市农业游两个省级片区，持续推进平山西柏坡、正定古城片区和京石高铁沿线美丽乡村示范线建设，其他县（市）区各建设一个示范片。着力抓好10个以上中心村、10个以上旅游村建设。完善农村基础设施，改造县乡公路400公里，新增变电容量169万千伏安，新建输电线路236公里。努力增加农民收入。推进农民工市民化，鼓励有稳定就业和收入的农业人口进城落户，让新市民共享城市公共服务。完善农产品价格保护和农业补贴政策，健全农村征地补偿制度，不断提高补偿标准，增加农民土地增值收益。努力拓宽农民增收渠道，确保农民收入增幅高于全市居民平均水平。加大脱贫攻坚力度。以四个国家级贫困县为重点，完善市县乡脱贫责任制，建立年度脱贫攻坚报告和督查问责机制。加强贫困地区交通、水利、电力、信息等基础设施建设，支持每个贫困县打造一个现代农业产业园区，培育设施蔬菜、优质林果、特色养殖和手工业等脱贫产业。支持贫困地区农民工返乡创业，每个贫困县至少建立一个返乡创业园。深入实施山区教育扶贫工程，改善贫困地区办学条件和教育质量，确保贫困家庭劳动力至少掌握一门致富技能。加大政策脱贫力度，将贫困人口全部纳入重特大疾病救助范围，把完全或部分丧失劳动能力的贫困人口全部纳入农村低保；引导资金、土地、人才、技术等要素向贫困地区聚集，支持贫困县重点项目建设。推进易地搬迁安置，完善搬迁后续扶持政策，改善搬迁群众生产生活条件，年内确保完成6.2万贫困人口稳定脱贫。

（六）以做强做美省会、做大做优县城为目标，加快新型城镇化步伐。抓住省委、省政府出台支持省会建设发展实施意见的重大机遇，修编石家庄市城市总体规划，集中实施一批重大城建项目，完善省会功能，拓展发展空间，提高城市品位。完善交通路网体系。启动石济客专火车东站及广场建设，确保2017年“五一”前竣工。抓好南二环东延西拓、和平路高架西延、裕华路西延、107国道、307辅线改造工程，确保年底前建成通车。按照一级公路标准，启动衡井线、新赵线、338国道、234国道新改建工程，形成省会新的绕城公路。加快轨道交通建设，确保1号线一期和3号线一期工程首开段年底前达到联调联试和工程验收条件，开工建设2号线一期和1、3号线一期两边段工程。加快推进槐安路与友谊大街匝道、槐安路东二环立交桥工程，建设华南路、明珠街等20条支路，完善新火车站区域路网。加快公交都市建设步伐，启动体育大街快速公交BRT2线建设，新建改造30座港湾式公交停靠站。提升城市载体功能。高标准抓好中山路综合改造，打造具有省会特色的繁华大道。出台供热规划，理顺供热管理体制，抓好电厂余热入市工程建设，新增市区供热面积1400万平方米。启动滹沱河市区段16公里生态景观建设工程，对河流两侧2700万平方米范围实施高水平绿化美化。推进龙泉湖公园、滹沱河滨水公园、体育公园等5个公园建设，打造省会最具规模的风景区。加强城市地下管廊建设，推进城市雨污分流工程，抓好黑臭水体治理工作。积极推进海绵城市建设，新开工项目全部按照海绵城市要求实施。大力发展节能建筑，启动1个超低能耗被动式建筑试点，完成20万平方米既有建筑节能改造。加大老旧小区基础设施改造提升力度，完成387个老旧小区改造任务，解决100个老旧小区无物业管理和100个老旧小区3层以上吃水难问题。强力推进正定新区建设。把滹沱河北岸作为省会建设主战场，集中谋划实施41个、总投资431亿元的基础设施和功能性项目。加快启动图书馆、规划馆、青少年宫等5大场馆建设，力促碧水源集团河北总部、省二院分院、宝能医院等项目上半年开工，奥体中心年底竣工投用。加强正定古城保护，按期完成古城墙修复、阳和楼、南关村综合改造等重点工程，努力使正定古城成为一颗展示省会厚重历史文化的璀璨明珠。实施智慧城市建设工程。重点推进城市运营中心和云计算数据中心、智慧城市时空信息云平台建设，以及公共安全、智慧网格、智慧政务、智慧交通、智慧医疗等5项智慧应用项目，提升城市现代化管理水平。实施公共场所免费Wi-Fi服务工程，二环路以内和正定新区主要公共场所实现全覆盖。深入推进“以克论净”城市保洁模式，开展占道经营、门前三包、渣土治理、楼宇天线和露天炭火烧烤等五个专项行动，提升市区精细化管理水平。强力推进市区交通拥堵治理，通过实施堵点改造、完善道路配套设施、优化交通组织等综合措施，力争市区道路拥堵问题得到有效缓解。规范停车场管理，增加停车位数量，对市区公共停车场实现“六统一”管理和“智慧泊车”。加速县城扩容提质。深入实施县城建设三年攻坚行动，完善县城规划体系，推进经济社会发展规划、城乡建设规划、土地利用规划等“多规合一”。加快基础设施建设，力争年内所有县城实现集中供水、集中供热或清洁能源供热、污水垃圾达标处理。大力提升县城环境容貌，加快迎宾景观大道、标志性街道、特色街区、精品建筑的改造提升。支持晋州、高邑争创省级新型城镇化试点，支持晋州、正定申报国家级园林县城，支持行唐、灵寿、赵县、无极争创省级园林县城。坚持小城镇大战略，重点培育一批工业型、旅游型、历史文化型专业特色城镇，提升城镇集聚人口和产业的能力。

（七）以深化改革扩大开放为引领，增强经济发展活力。积极跟进落实中央、省出台的重点改革举措，提升改革开放的深度和广度，构建开放型经济新体制。更大力度推进重点领域改革。深化人事制度改革，在高新区实行全员岗位聘用管理制度。深化商事制度改革，全面推行“电子营业执照”，加强事中事后监管，完善社会信用体系。积极推进国有企业混合所有制改革，抓好股权激励和员工持股试点，力争完成保安服务公司等3家企业改制工作。深化财政金融体制改革，全面完成“营改增”改革，完善以绩效预算为引领的预算管理制度。大力推广PPP融资模式，积极引进社会资本参与城市基础设施建设。按照省要求，上半年提早完成公务用车制度改革。深化农村综合改革，重点抓好农村土地承包经营权确权登记颁证工作，力争确权登记面积达到80%以上。深化供销社综合改革，健全农村综合服务体系。更大力度推进双向开放。实施精准招商、以商招商、中介招商、集群招商，精心办好石洽会等系列招商引资活动，努力提高招商项目落地率。支持钢铁、水泥、机械装备、轻工纺织等产业开展国际产能合作，力争新增境外投资企业10家以上。全年实际利用外资增长6%。更大力度加快开发区建设。把开发区作为推进转型升级、开放发展的主战场，对全市25个省级开发区的经济发展、基础建设、生态环保等6项内容实施分类评价，建立有进有出的动态管理机制，对排名领先的开发区在建设用地指标、评先评优上给予奖励。大力实施品牌带动战略，创建具有地域特点、产业特色、开放特征的品牌园区，力争10个以上园区列入省级新型工业化示范基地。更大力度稳定对外贸易。着力抓好8个省级以上出口基地建设，大力培育出口品牌，鼓励企业开展境外商标注册、出口和宣传推广，努力扩大具有自主知识产权、科技含量高的产品出口规模。支持企业扩大先进技术装备、关键零部件以及重要原材料进口，全年外贸出口总值增长6%。

（八）以提高环境质量为核心，全面抓好生态保护和修复。坚持源头防控、终端管控、严格执法，持续推进生态环境治理。全力改善大气环境质量。制定三年退出全国大气质量重点监测城市后十位时间表、路线图。强力推进“压煤、抑尘、控车、减排、迁企、增绿”六大措施，开展散煤、焦化行业、露天矿山、道路车辆污染整治四大专项行动，对重点行业和20蒸吨以上燃煤锅炉安装自动监控设施，年内净削减煤炭50万吨；开展工业挥发性有机物治理，重点行业治理项目完成率达到80%；加大中心城区工业企业搬迁力度，推进常山纺织、欧意药业、中诺药业等企业搬迁，确保空气质量明显好于上年。加强水环境综合整治。实施滹沱河下游污染综合治理和洨河、汪洋沟水质提升工程，启动岗南、黄壁庄饮用水源地一级保护区隔离围挡工程，确保饮用水安全。加快南水北调配套工程建设，17座县级水厂全部投用，市区东北水厂、良村开发区水厂具备通水能力。抓好地下水超采综合治理试点市建设，编制地下水超采综合治理中长期规划，新增农业压采地下水能力8300万立方米。实施西北污水处理厂升级改造工程，确保出厂水质达到一级A标准。深入实施生态修复工程。重点抓好太行山生态绿化、矿山绿化、过境河流两岸造林、东部特色经济林等造林绿化项目，完成太行山区人工造林60万亩，封山育林120万亩，平原植树造林10万亩，全市森林覆盖率达到38.5%。加强山水林田湖综合治理，制定土壤污染防治计划，稳步有序治理农业面源污染。完善西部生态区域补偿机制，打造生态、富裕、和谐的西部生态区。加强环境保护监督管理。完善与财政挂钩的空气质量考核奖惩制度，以环保网格化管理、行政处罚、排污收费、信息公开为手段，突出重点区域、重点企业环境监管。严格落实生态环境督办问责制度，开展生态环境、自然资源责任审计。对排污企业实行在线监测，依法严厉打击环境违法行为。

（九）以增强人民群众幸福感为根本，着力保障和改善民生。坚持守住底线、突出重点、加大投入，让人民群众过上更加幸福美好的生活。努力扩大就业再就业。认真落实推进“双创”政策措施，实施更加积极的就业创业政策，鼓励科技人才、高校毕业生、退役军人自主创业，以创业带动就业，全年城镇新增就业9.52万人。完善社会保障体系。推进城镇职工和居民基本医疗保险异地就医直接结算，城镇职工参加基本养老保险人数达到217.5万人、城镇职工和居民基本医疗保险参保人数达到284万人。开工建设棚户区改造安置住房2.3万套，农村危房改造3500户。新建社区综合养老服务中心10家，新增护理型养老床位1000张。开展特殊群体帮扶工程，切实落实好计划生育特殊家庭医养扶一体化、困难残疾人生活补贴和重度残疾人护理补贴等帮扶政策。提高医疗保障水平。巩固完善基本药物制度，全面提升基层医疗机构服务能力，探索建立分级诊疗模式。推进“国家中医药综合改革试验市”工作，重点抓好50所中医药特色乡镇卫生院建设。进一步提高新农合保障水平，新农合参合率达到95%以上。实施全面两孩政策，促进人口长期均衡发展。优先发展教育事业。深入实施学前教育第二期行动计划，创办30所普惠性幼儿园。创新小学生免费托管服务方式，在主城区40所小学实现托管服务。抓好国家中小学综合质量评价、特殊教育综合改革示范区工作，继续实施优质教育资源提升扩充工程。加快发展现代职业教育，采取兼并、托管、合作办学等模式，重点引进京津高职教育优质资源，培养高素质劳动者和高等技术技能人才。健全文化服务体系。加快文化设施建设，确保霞光大剧院10月底建成投用。加强基层文化站建设，完善基层公共文化服务网络，对100个社区文化活动室和文化广场进行提档升级。继续开展“彩色周末”、专业艺术院团下基层演出、公益电影放映等文化惠民活动。制定全民健身行动计划，更新健身路径170条。推进社会治理创新。加快食品药品可追溯制度建设，确保人民群众饮食用药安全。强力排查整治安全生产隐患，坚决遏制重特大事故发生。切实规范社会和民间组织的融资行为，坚决守住不发生系统性和区域性金融风险的底线。完善信访绿色通道制度，方便群众表达诉求。深化平安省会建设，继续实施“天网”工程，完善社会治安防控体系，严厉打击各种违法犯罪活动。加强国防教育和国防后备力量建设，深入开展双拥共建工作。积极做好民族宗教、外事侨务、人防档案、气象防震、妇女儿童、残疾人等各项工作，促进社会事业全面进步。

四、切实加强政府自身建设

面对经济发展新常态、率先发展新任务，我们必须加快转变政府职能，加强创新型、实干型、服务型、法治型、廉洁型政府建设，为率先全面建成小康社会提供坚强保障。

（一）解放思想，开拓进取，建设创新型政府。牢固树立和贯彻落实“五大发展理念”，坚决破除制约发展的思想观念、制度藩篱，实现思想再解放、认识再提升。坚持高标准、严要求，力求各项工作都做到细致、精致、极致。增强互联网思维，加大政务信息公开力度，为群众提供及时、便捷、高效服务。突出问题导向，创新发展路径，创新政策措施，努力在转换增长动力、优化经济结构、补齐发展短板等方面取得突破性进展。

（二）敢于担当，恪尽职守，建设实干型政府。坚持真干实干苦干巧干，强化省会意识、率先意识，一切工作都按照在全省创一流、当典范、树标杆的原则进行谋划、部署和推动，确保各项工作走在全省前列。大力开展“问计省直”活动，最大限度争取省直部门支持。进一步完善工作推进机制，建立健全末位淘汰机制、捆绑考核机制、督查要账机制。强化功成不必在我的发展理念，多做一些打基础、利长远、惠民生的实事、好事，以十年一剑、滴水穿石的执着精神推动发展。

（三）转变作风，提高效率，建设服务型政府。把“马上就办、办就办好”作为基本准则，把“夙兴夜寐、激情工作”作为新常态。加快转变政府职能，深化“放管服”改革，进一步精简和规范行政审批事项。坚决砍掉繁琐手续，缩短办理时限，打通服务企业、联系群众的“最后一公里”。积极推行政府购买服务制度，为人民群众提供更多更好的公共产品和服务。大力改进会风文风，深入基层调查研究，真正把精力用在抓落实上、把功夫下在见实效上。

（四）依法行政，照章办事，建设法治型政府。坚持依宪施政、依法行政，做到机构、职能、权限、程序、责任法定化。健全政府法律顾问制度，完善重大事项集体决策机制，确保政府的一切工作在法律的框架下进行。自觉接受市人大的法律监督、市政协的民主监督和社会公众的舆论监督，保障和支持审计、监察等部门依法独立行使监督权。完善行政执法程序，规范行政执法行为，做到严格执法、公正执法、文明执法。

（五）严守纪律，艰苦奋斗，建设廉洁型政府。严守政治纪律和政治规矩，认真践行“三严三实”要求，严格落实中央“八项规定”精神，坚持不懈纠正“四风”。始终牢记“两个务必”，坚持勤俭节约办事、集中财力办大事。严格落实党风廉政建设“两个责任”和“一岗双责”，加强惩治和预防腐败体系建设，坚持无禁区、全覆盖、零容忍，强化对人权、事权、财权的制度约束，努力做到干部清正、政府清廉、政治清明。

各位代表！率先全面建成小康社会的蓝图已经绘就，加快转型升级、跨越赶超的号角催人奋进。让我们紧密团结在以习近平同志为总书记的党中央周围，在省委、省政府和市委的正确领导下，紧紧依靠全市人民，解放思想，抢抓机遇，奋发作为，加快转型升级、跨越赶超、建设幸福石家庄步伐，为在全省率先全面建成小康社会而努力奋斗！
